# Revealing that artificial reproduction promotes increased genetic diversity between generations in *Carpinus putoensis*

**DOI:** 10.3389/fpls.2025.1494694

**Published:** 2025-02-27

**Authors:** Dingsheng Li, Kai Gao, Yeping Chen, Haojie Gao, Haiming Huang, Bo Ye, Lei Shi, Haina Yu, Ying Zhao

**Affiliations:** ^1^ Zhoushan Academy of Forestry, Zhoushan, Zhejiang, China; ^2^ Research Institute of Subtropical Forestry, Chinese Academy of Forestry, Hangzhou, Zhejiang, China; ^3^ Zhoushan Forest Farm of Zhejiang, Zhoushan, Zhejiang, China

**Keywords:** *Carpinus putoensis*, genetic diversity, mating system, SSR, endangered plants

## Abstract

**Introduction:**

*Carpinus putoensis*, an endemic species of Putuo Island in the Zhoushan Archipelago, Zhejiang Province, China, is listed as critically endangered (D1) in the 1998 World List of Threatened Trees.

**Methods:**

Using 15 pairs of SSR primers markers, 143 individuals from three population generations were analyzed, generating 193 alleles.

**Results and Discussion:**

The average number of alleles (*N_a_*) was 12.9, ranging from 4 to 28, while the average effective number of alleles (*N_e_*) was 4.900, with a range of 1.649 to 11.602. The multilocus outcrossing rate (*t_m_*) was 1.000, and the single-locus outcrossing rate (*t_s_*) was 0.871, ranging from 0.751 to 0.920 across the families studied. The difference between *t_m_* and *t_s_* (0.129) and the positive biparental inbreeding coefficients (0.080 to 0.249) indicate the presence of inbreeding. Moreover, an increase in *N_a_* and Ne was observed across generations, from 6.400 and 2.838 in the F1 generation to 9.200 and 4.228 in the F3 generation, respectively. These results highlight the need for artificial interventions to increase population size and improve genetic diversity, which are critical factors for the conservation and recovery of *C. putoensis*.

## Introduction

*C. putoensis* is endemic to Putuo Island of the Zhoushan Archipelago, Zhejiang Province, and is designated as a first-tier protected plant species in China (Meng et al., 2004; Li et al., 2010; Qin and Zhao, 2017; Sheng et al., 2021). It is classified as “Critically Endangered” on the International Union for Conservation of Nature (IUCN) Red List(https://www.iucnredlist.org/species/32303/2813038). The population of *C. putoensis* has experienced a precipitous decline due to historical human activities such as deforestation and land reclamation, with the population being reduced to a single mature individual at one time. No more wild individuals have been found since the early 1930s (Sheng and Zhu, 2018). Subsequent to these events, Chinese scientists have implemented scientific conservation strategies and artificial propagation techniques, which have led to a partial restoration of the species’ population. Conservation efforts include the safeguarding of the solitary wild specimen and the augmentation of the population through artificial pollination and seed cultivation methods. Presently, the first and second generations, resulting from natural pollination, have been successfully bred from seeds collected artificially from the original wild progenitor. In light of the endangered status of *C. putoensis*, researchers have undertaken a series of research initiatives focusing on its biological characteristics, growth processes and patterns, as well as its reproduction, cultivation, and *ex situ* conservation techniques (Li et al., 2010). These studies aim to understand the species’ ecological requirements better, enhance its propagation success, and develop strategies for its conservation and sustainable use. However, little is known about genetic diversity and mating system of *C. putoensis*.

Genetic diversity is a pivotal attribute in reflecting the genetic structure of populations, as it indicates the richness of existing genotypes within a species and elucidates the interplay between genetic diversity and factors such as geographical distribution and ecological environment (Addisalem et al., 2016; Yoder et al., 2018; Bessega et al., 2019; Curry et al., 2021; Maroso et al., 2021). The mating system in plants is a sexual mechanism that sustains genetic linkage between generations, dictating the genotype distribution and demographic dynamics of the offspring (including effective population size, sex ratio, and the extent of random mating), which is intricately tied to the genetic dynamics within populations and aids in comprehending the genetic ramifications of reduced population size (Neel, 2002; Spielman et al., 2004). Rare and endangered plants face habitat fragmentation, dwindling wild population numbers, and challenges in natural regeneration due to many factors, including biological characteristics, climate change, interspecific competition, and anthropogenic disturbances (Feeley and Silman, 2009; Jia et al., 2020; Li et al., 2022; Antonelli et al., 2023). Research into the genetic structure of endangered populations, their mating systems, and their interactions with the environment can uncover the mechanisms leading to species endangerment and decline, analyze the patterns of genetic diversity across parent and offspring generations, and hold significant implications for guiding the recovery efforts of vulnerable populations. Due to differences in plant density, flowering plant density, population size, pollinators, and floral morphology (Breed et al., 2012; Whitehead et al., 2018), there are significant differences in mating systems within and between populations. Additionally, there are temporal and spatial differences in the mating systems of different plants within the same species and even among the fruits of a single individual (Mathiasen et al., 2007; Tamaki et al., 2009; Silva et al., 2011).

With the rapid advancement of molecular biology, molecular markers such as isozymes, SSR (Simple Sequence Repeat), AFLP (Amplified Fragment Length Polymorphism), and RAPD (Random Amplified Polymorphic DNA) have been extensively utilized in the analysis of mating systems and genetic diversity. Co-dominant loci typically provide more information per locus than dominant ones (Ashley, 2010). SSR molecular markers are considered the most suitable for studying mating systems and genetic diversity due to their high degree of polymorphism, abundance, and random distribution throughout the genome, stable amplification, and co-dominant Mendelian inheritance in both neutral and multi-allelic forms (Yang et al., 2016; Abbasov et al., 2019). Their successful application in elucidating the genetic relationships of certain tree species has been well documented (Yang et al., 2017; Lopez-Villalobos and Eckert, 2018; Vinson et al., 2018).

In this study, 15 pairs of SSR primers were obtained through the screening of transcriptome data, and a systematic analysis was conducted on the genetic diversity and kinship relationships of the first, second, and third generations of the rare and endangered plant *C. putoensis*. Furthermore, the mating systems of six families of *C. putoensis* were assessed. This study is expected to provide a theoretical basis for the formulation of conservation strategies, breeding of improved varieties, and further development and utilization of *C. putoensis*.

## Materials and method

### Sample collection

**Figure 1:**
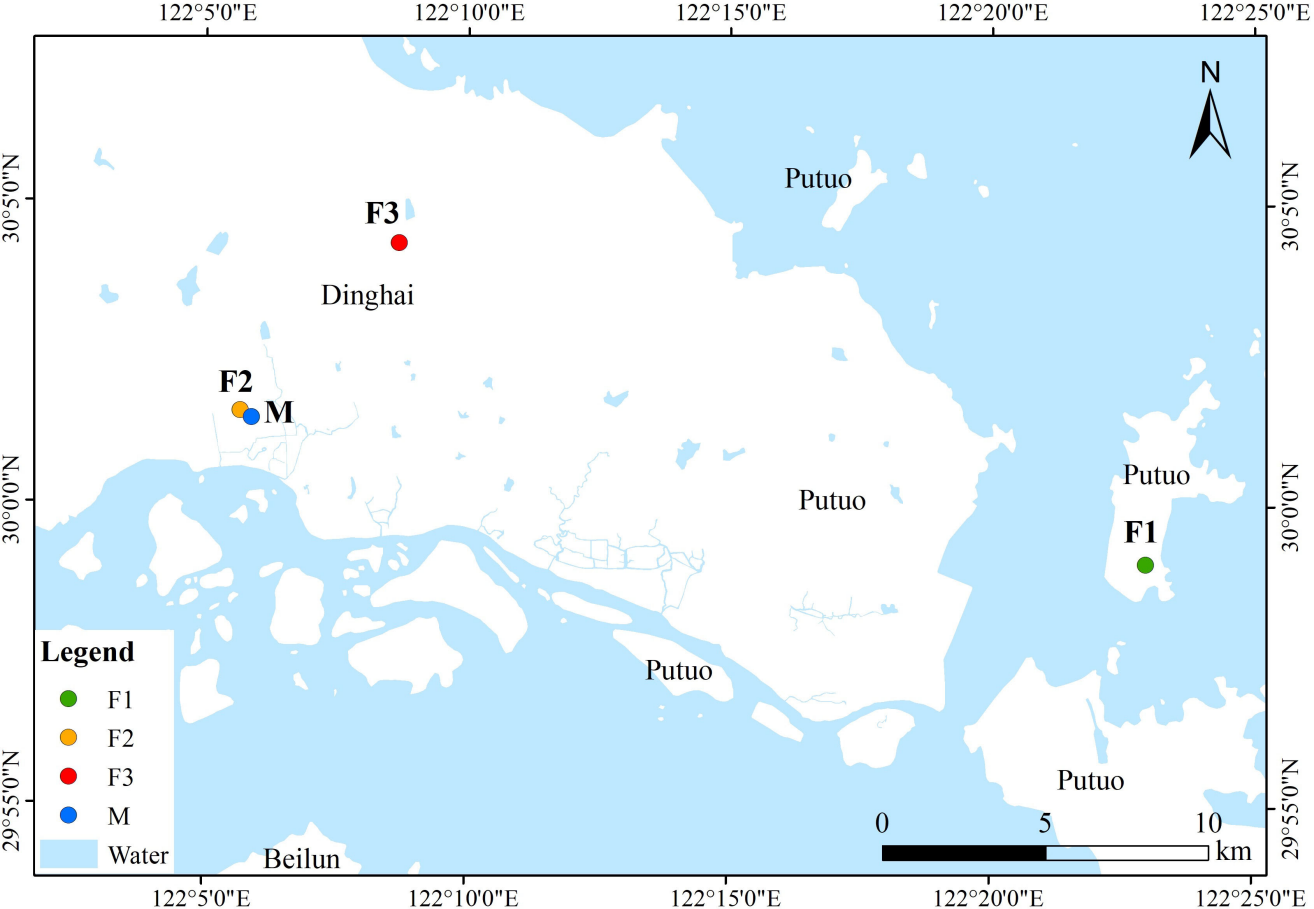
Locations of *C. putoensis* populations in China. Note: F1: first filial generation; F2: second filial generation; F3: third filial generation; M: six family lineages.

In the early 1960s, a survey on the northwest slope of Buddha’s Summit Mountain in Putuo Island, Zhoushan City, identified a sole surviving *C. putoensis* mother tree, around 200 years old, with no natural regeneration of seedlings observed in its vicinity to this day. The Zhoushan Academy of Forestry collected seeds from this tree in 1981 and artificially propagated 79 first filial generation (F1) seedlings in the nursery, which were then transplanted to the tea garden area of Buddha’s Summit Mountain. In 2003, seeds from the F1 generation were harvested, and over 1000 second filial generation (F2) seedlings were artificially propagated and transplanted to the Zhoushan Academy of Forestry and Wofo Mountain Villa. In 2022, natural pollination within the F2 population at Wofo Mountain Villa yielded 230 third filial generation (F3) seedlings. In 2014, six individuals with robust growth were selected from the F2 generation population at the Zhoushan Academy of Forestry site. Seeds from their half-sibling families were utilized for the cultivation of seedlings, and a total of six half-sibling families were analyzed in the mating system analysis, with family sizes ranging from 26 to 31 individuals. The 47 individuals of the F1 generation are sequentially numbered from PT1-1 to PT1-47, while the 48 individuals of the F2 generation are numbered from PT2-1 to PT2-48, and those of the F3 generation from PT3-1 to PT3-48. A total of 312 individuals of *C. putoensis* were collected, of which 143 were from the 3-generation population and 169 were from six family lineages in Zhoushan, Zhejiang, China (**Figure 1**, **Table 1**). The three generations were used to estimate all genetic parameters, except for the mating system, which was based on six lineages from the second generation.

**Table 1:** Geographical information of 3 generations of *C. putoensis*.

Populations	Sample individual number	Longitude	Latitude	Altitude (m)	Site
F1	47	E122°22′58″	N29°59′02″	65	Putuo Mountain Scenic Spot, Zhoushan, Zhejiang, China
F2	48	E122°05′41″	N30°01′31″	10	Zhoushan Forestry Academy of Zhejiang, Zhoushan, Zhejiang, China
F3	48	E122°05′21″	N30°06′18″	57	Wofo Mountain Villa, Zhoushan, Zhejiang, China
Z3	31	E122°05′54″	N30°01′24″	10	Zhoushan Forestry Academy of Zhejiang, Zhoushan, Zhejiang, China
Z5	30	E122°05′54″	N30°01′24″	10	Zhoushan Forestry Academy of Zhejiang, Zhoushan, Zhejiang, China
Z6	31	E122°05′54″	N30°01′24″	10	Zhoushan Forestry Academy of Zhejiang, Zhoushan, Zhejiang, China
Z7	27	E122°05′54″	N30°01′24″	10	Zhoushan Forestry Academy of Zhejiang, Zhoushan, Zhejiang, China
Z9	26	E122°05′54″	N30°01′24″	10	Zhoushan Forestry Academy of Zhejiang, Zhoushan, Zhejiang, China
Z10	30	E122°05′54″	N30°01′24″	10	Zhoushan Forestry Academy of Zhejiang, Zhoushan, Zhejiang, China

F1: first filial generation; F2: second filial generation; F3: third filial generation; Z3, Z5, Z6, Z7, Z9, Z10: six family lineages.

### Transcriptome data acquisition

Total RNA was isolated from leaves of each individual tree using a commercial RNA extraction kit (Aidlab, Beijing, China). The quality and integrity of RNA samples were assessed using agarose gel electrophoresis and a NanoDrop spectrophotometer. Subsequently, equal amounts of RNA from each tissue were pooled to create a representative RNA sample for each individual.

High-throughput RNA sequencing (RNA-Seq) was conducted using Illumina sequencing technology, with an average sequencing depth of more than 50×, and saturation tests were performed to ensure comprehensive coverage. cDNA libraries were constructed from the pooled RNA samples, and sequencing was performed with paired-end reads. The sequencing depth was carefully controlled to ensure comprehensive coverage of the transcriptome.

### Data preprocessing

Raw RNA-Seq data underwent preprocessing steps to ensure data quality. Adapter sequences and low-quality bases were removed using standard bioinformatics tools. High-quality paired-end reads were retained for further analysis.

### *De novo* transcriptome assembly

*De novo* assembly of the transcriptome was conducted using advanced assembly algorithms such as Trinity (Grabherr et al., 2011) or SOAPdenovo-Trans (Xie et al., 2014). This process aimed to create a non-redundant reference transcriptome for subsequent SSR discovery.

### Identification of SSR loci

The assembled transcriptome sequences were systematically screened for the presence of simple sequence repeat (SSR) motifs using specialized software MISA (Thiel et al., 2003). SSR detection criteria included a minimum repeat unit size of 2, 3, 4, 5, 6, and 7 nucleotides.

### SSR characterization

Characterization of the identified SSR loci involved documenting information about the repeat motif type, repeat length, and the number of repeats. Details regarding the flanking regions, including primer sequences, were extracted for primer design.

Primer Design:

Primer pairs were designed for the selected SSR loci using Primer3 (Untergasser et al., 2012). Careful consideration was given to factors such as optimal annealing temperature, primer length, and the absence of secondary structures. Priority was given to SSR markers with an adequate number of repeats and even distribution across the transcriptome.

### Validation and amplification

The synthesized primer pairs were empirically validated for their amplification success and polymorphism in a representative subset of *C. putoensis* individuals. Polymerase chain reaction (PCR) amplifications were carried out, and the resulting amplicons were analyzed using gel electrophoresis or capillary electrophoresis. A total of 15 SSR primer pairs with clear band patterns and high polymorphism were selected (**Table 2**).

**Table 2:** Information of 15 pairs of primers used for SSR marker analysis on populations of 3 generations of *C. putoensis*.

Primer	Sequence (5’→3’)		Repeated motif	Fragment length/bp	Annealing temperature/°C
	Forward primer	Reverse primer			
L3	TGTAATTTTTAATGAGAAACTCAATCG	ATATGTCAACACAAAGGGAGGG	(AT)_16_	87-126	54
L7	ATGTGATCCACGTGACGC	GCCTCAAAATTAAAACAGTGTCAT	(AT)_14_	94-128	54
P1	GAAGAATGGAAGGTGCTCCA	TACTCTCCAAGCAGCACTCG	(TGC)_5_	282-330	58
P3	TGTTCTTGAGCTGAACACGG	GCCAGCACGCAGAAGTAAAT	(GA)_9_	166-208	58
P5	TGTTGTTGTTTGGGGACAGA	TCGGCTTTCACTCTTTGAGTC	(AG)_6_	253-287	58
P7	TGCTTCTCTCCCAAAATTCA	GAGAGGAAGCAGAAACGGTG	(TC)_6_	157-183	58
P8	GTGCTTCGGCTCTTGAAGTC	TGTAGTCGGTGGAAGCTCCT	(CTC)_7_	188-258	58
P9	CAATATCACGTTCCCCTGCT	CCGTGGTTAAGACCAGCATT	(GAA)_5_	255-294	58
P11	TTTGGACTTTACTTTTCTGGGG	CGGCCTCTTCCTCCTCTATT	(GAT)_5_(GTT)_5_	187-220	58
P12	ACAGCAGGGGTGTTCGCTAC	GAAATGGCTCCATTGTCGTT	(AGT)_5_	258-348	58
P13	ACGTACTACGCATCACAGGG	GATGAATCCGGCTTTGACAT	(AG)_10_	190-294	58
P14	CTCACTCTCTCGCAGGCAG	AAGATCGTGGTCTGTGGAGG	(TC)_6_	196-232	58
P15	ATGTCTGAACGGAGTGGCTT	CGCCCGACTTATTCTCTGAA	(A)_14_	200-248	58
P16	AGTTCGGGTGGCCTACTTTT	ACCACCAACGCCACAACT	(TGG)_6_	121-205	58
P17	AATGGCCGAGAGGATTTCTT	ATGGATCCCCACATTTTTCA	(CAA)_7_	190-232	58

Amplification was performed in a 25µL reaction mixture, containing 1µL of DNA template (10ng/µL), 12.5µL of 2 × Taq Plus Master Mix (Vazyme, China), 9.5µL of ddH2O, 1µL of reverse primer(1µM), and 1µL of forward primer (1µM). The PCR program was as follows: pre-denaturation at 94°C for 3 min, followed by 35 cycles of denaturation at 95°C for 15 s, annealing at 58°C for 15 s (58°C for all primers, and 54°C specifically for L3 and L7 primers), and extension at 72°C for 30 s, with a final extension at 72°C for 5 min. The PCR products were analyzed by capillary electrophoresis in an Qsep100 DNA Fragment Analyzer (Bioptic, New Taipei City, China). The SSR allele size was determined by Q-Analyzer software (Bioptic, New Taipei City, China).

### Genetic diversity Analysis:

GenALEx 6.5 software (Peakall and Smouse, 2012) was used to analyze the genetic diversity of the *C. putoensis* 3 generations population, such as the number of alleles (*N_a_*), the effective number of alleles (*N_e_*), observed heterozygosity (*H_o_*), expected heterozygosity (*H_e_*), Fixation index (*F_i_*) and Shannon’s information index (*I*). *F_i_* was calculated according to the following formula: *F_i_* =1- *H_o_*/*H_e_*.

CERVUS 2.0 software (Kalinowski et al., 2007) was used to perform Hardy-Weinberg equilibrium test on the 3 generations population of *C. putoensis*, with a minimum expected frequency of 5. Using Ye’s continuity correction and Bonferroni correction, the polymorphism information content (*PIC*) of microsatellite loci was obtained.

Calculate allelic richness (*AR*) and private allelic richness (*PA*) using HP-RARE1.1 software (Kalinowski, 2005).

Using FSTAT 2.9.3 software (Goudet, 1995) to detect the differences in genetic diversity parameters among the first, second, and third generation populations (1000 simulations), and calculate the inbreeding coefficient (*Fis*) of the 3 generations population.

### Genetic Kinship Analysis of Progeny:

The genetic distance (GS) for all individuals across the three generations of *C. putoensis* were determined, data visualization was achieved using the TB Tool software (Chen et al., 2020). A dendrogram was constructed using an unweighted pair-group method with arithmetic means (UPGMA) algorithm based on Nei’s genetic distance among populations using the NTSYS version 2.1 software (Rohlf, 1992). The circular dendrogram was constructed using MEGA 11 software (Tamura et al., 2021).

### Mating system Analysis:

The mating system parameters were estimated both on the level of individual families and at population level using known maternal parents. The parameters included multi-locus (*t_m_*) and single locus outcrossing rate (*t_s_*), biparental inbreeding (*t_m_*-*t_s_*), and correlation of outcrossed paternity (*r_p_*) [including multi-locus paternity correlation (*r_p(m)_*) and single-locus paternity correlation (*r_p(s)_*)], which were calculated based on the multi-locus mixed-mating model and the estimation procedure described in Ritland and Jain (1981). These parameters were estimated from the progeny samples using the MLTR v3.4 software (Ritland, 2002) and the expectation-maximization (EM) algorithm with the following default parameters: t = 0.9, FM = 0.1, rt = 0.1, rp = 0.1, calculated with 95% confidence interval. The standard errors were calculated with 1000 bootstrap repeated sampling within each investigated family and were used to assess whether the mating parameters were significantly lower than one or greater than zero.

In the above analyses, mating system parameters (*t_m_*, *t_s_*, *r_p_*) were used to estimate other demographic and genetic parameters (*N_ep_*). The effective number of pollen donors over maternal trees was calculated from the paternity correlation by the equation *N_ep_* = 1/*r_p (m)_*.

## Results

### Genetic diversity

15 pairs of SSR primers used to generate 193 alleles from 143 individuals of 3 generations population of *C. putoensis* (**Table 3**). The results showed 12.9 alleles per locus (ranging from 4 (L7, P17) to 28 (P13)). The *N*_e_ for each locus ranged from 1.649 (L7) to 11.602 (P13), with an average of 4.900. The *H_o_* for each locus varied from 0.084 (L3) to 1.000 (P15), with an average of 0.577, whereas the *H*_e_ ranged from 0.394 (L7) to 0.914 (P13), with an average of 0.729. The *I* ranged from 0.690 (L7) to 2.770 (P13), with an average of 1.727. The *PIC* (0.341 (L7) – 0.908 (P13)) were detected, with an average of 0.697, indicating a high level of polymorphism among the primers. The *Fi* of the corresponding loci of L7, P1, P8, P15, and P16 primers in 15 pairs of SSR primers is less than 0, indicating the presence of heterozygous excess within the *C. putoensis* population.

**Table 3:** Genetic parameters of loci corresponding to 15 pairs of primers used for SSR marker analysis on populations of 3 generations of *C. putoensis*.

Primer	*N*_a_	*N*_e_	*H*_o_	*H*_e_	*I*	*PIC*	*F_i_*
L3	12	2.528	0.084	0.604	1.338	0.569	0.861
L7	4	1.649	0.503	0.394	0.690	0.341	-0.279
P1	12	5.807	0.986	0.828	1.975	0.807	-0.191
P3	13	4.282	0.552	0.766	1.876	0.748	0.279
P5	16	7.926	0.469	0.874	2.330	0.862	0.464
P7	9	4.422	0.098	0.774	1.654	0.738	0.873
P8	18	6.980	0.972	0.857	2.239	0.842	-0.135
P9	12	6.471	0.818	0.845	2.058	0.828	0.032
P11	6	2.347	0.126	0.574	1.076	0.515	0.781
P12	22	4.850	0.720	0.794	2.134	0.779	0.093
P13	28	11.602	0.874	0.914	2.770	0.908	0.043
P14	9	2.611	0.371	0.617	1.235	0.569	0.399
P15	18	5.858	1.000	0.829	2.054	0.809	-0.206
P16	10	4.088	0.979	0.755	1.624	0.719	-0.296
P17	4	2.044	0.098	0.511	0.847	0.427	0.808
Mean	12.900 ± 1.718	4.900 ± 0.690	0.577 ± 0.093	0.729 ± 0.039	1.727 ± 0.152	0.697 ± 0.171	0.235 ± 0.113

*N*_a_, number of alleles; *N*_e_, effective number of alleles; *H*_o_, observed heterozygosity; *H*_e_, expected heterozygosity; *I*, Shannon’s information index; *PIC*, polymorphism information content; *F_i_*, Fixation index.

The genetic diversity of 3 C*. putoensis* generations population was shown in **Table 4**. The average *N_a_* was 7.822, and it varied from 6.400 for the F1 generation population to 9.200 for the F3 generation population, whereas the average *N_e_* was 3.497, ranging from 2.838 for the F1 generation population to 4.228 for the F3 generation population.

**Table 4:** Comparison on genetic diversity of populations of 3 generations of *C. putoensis*.

Populations	*N*_a_	*N*_e_	*H*_o_	*H*_e_	*I*	*F_i_*	*A_R_*	*P_A_*	*F_is_*
F1	6.400 ± 1.073	2.838 ± 0.366	0.565 ± 0.107	0.525 ± 0.074	1.124 ± 0.171	0.003 ± 0.114	6.400 ± 4.154	1.340 ± 1.403	-0.065 ± 0.441
F2	7.867 ± 1.158	3.424 ± 0.479	0.563 ± 0.094	0.627 ± 0.053	1.366 ± 0.152	0.172 ± 0.134	7.840 ± 4.464	1.400 ± 1.982	0.114 ± 0.517
F3	9.200 ± 1.114	4.228 ± 0.526	0.603 ± 0.084	0.700 ± 0.043	1.578 ± 0.144	0.121 ± 0.114	9.160 ± 4.288	2.590 ± 1.909	0.150 ± 0.441
Mean	7.822 ± 0.652	3.497 ± 0.274	0.577 ± 0.054	0.617 ± 0.035	1.356 ± 0.092	0.103 ± 0.069	7.800 ± 1.380	1.777 ± 0.705	0.066 ± 0.115

*N*_a_, number of alleles; *N*_e_, effective number of alleles; *H*_o_, observed heterozygosity; *H*_e_, expected heterozygosity; *I*, Shannon’s information index; *F_i_*, Fixation index; *A_R_*, allelic richness; *P_A_*, private allelic richness; *F_is_*, inbreeding coefficient.

Mean value of observed heterozygosity (*H_o_* = 0.577) were less than that of expected heterozygosity (*H_e_* = 0.617). The mean value of *Fi* was 0.099, ranging from 0.003 for the F1 generation population to 0.172 for the F2 generation population. The *I* varied from 1.124 for the F1 generation population to 1.578 for the F3 generation population, with a mean of 1.356.

Overall, the genetic diversity level of the 3 C*. putoensis* generations population is relatively high, and with the increase of generations, the genetic diversity level of the population also shows higher performance. The *H_o_* value of the F1 generation population is greater than the *H_e_* value, while the *H_o_* value of the F2 and F3 generation populations is less than the *H_e_* value. The *Fis* of the F1 generation population is less than 0, while the *Fis* value of the F2 and F3 generation populations is greater than 0, indicating an excess of heterozygotes in the F1 generation population and a loss of heterozygotes in the F2 and F3 generation populations.

In addition, the *H_o_* value of the F2 generation population is slightly lower than that of the F1 generation population, while the *H_o_* value of the F3 generation population is slightly higher than that of the F2 generation population. Although there are no significant differences among the three generations (**Supplementary Tables S1, S2**), this indicates a decrease in the actual heterozygous individual plant ratio in the F2 generation population compared to the F1 generation population, and an increase in the actual heterozygous individual plant ratio in the F3 generation population compared to the F2 generation population.

### Genetic kinship analysis of progeny populations in *C. putoensis*

**Figure 2:**
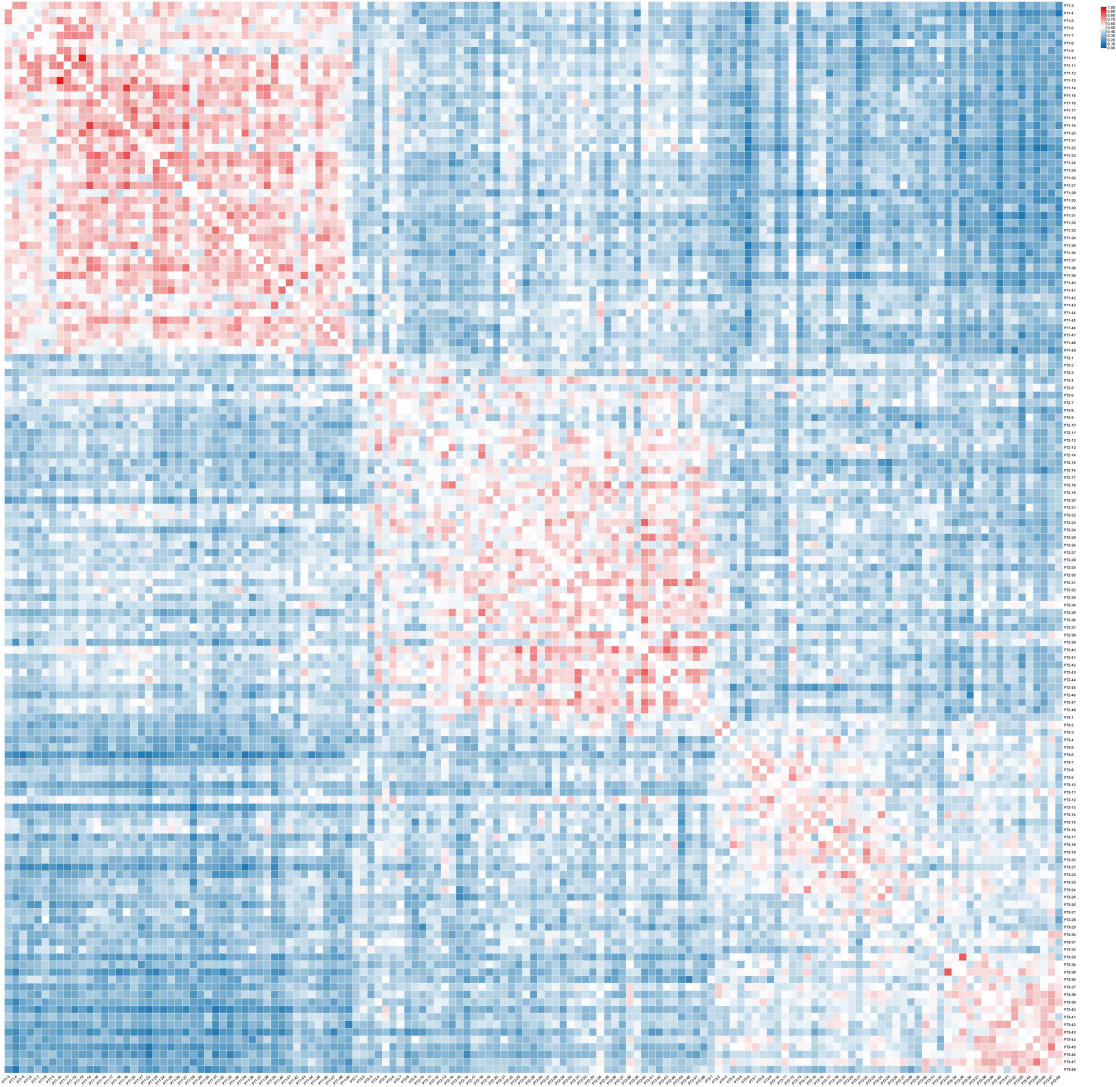
The genetic similarity coefficient of the 3 C*. putoensis* generations population.

The genetic similarity coefficient (GS) between 143 sample plants of the 3 C*. putoensis* generations population is 0.087~0.952 (**Figure 2**). Among the first-generation samples of *C. putoensis*, PT1-13 has the closest genetic relationship with PT1-10 (GS=0.952), while PT1-42 has the farthest genetic relationship with PT1-22 (GS=0.300). Within the second-generation samples, PT2-43 has the closest genetic relationship with PT2-40 (GS=0.818), whereas PT2-48 is genetically most divergent from PT2-3 (GS=0.222). In the third-generation samples, PT3-35 and PT3-33 are genetically most closely related (GS=0.833), while PT3-48 and PT3-28 are the most genetically distant (GS=0.227). When comparing the first and second generations, PT1-44 and PT2-34 display the highest genetic similarity (GS=0.638), whereas PT1-31 and PT2-39 present the lowest genetic similarity (GS=0.167). Between the second and third generations, PT2-38 and PT3-2 are identified as having the most proximal genetic relationship (GS=0.682), in contrast to PT2-46 and PT3-36, which exhibit the most divergent genetic profiles (GS=0.133). In comparison between the first and third generations, PT1-38 has the closest genetic relationship with PT3-16 (GS=0.583), while PT1-31 has the farthest genetic relationship with PT3-40 (GS=0.087). The heatmap results indicate that the genetic relationships among individuals within the first, second, and third generations of *C. putoensis* are relatively close, with a gradual decrease in genetic proximity observed across successive generations.

The x-axis and y-axis represent all individuals of the first, second, and third generations arranged in sequence. Red indicates higher GS, while blue indicates lower GS, with GS values ranging from 0.087 to 0.952.

**Figure 3:**
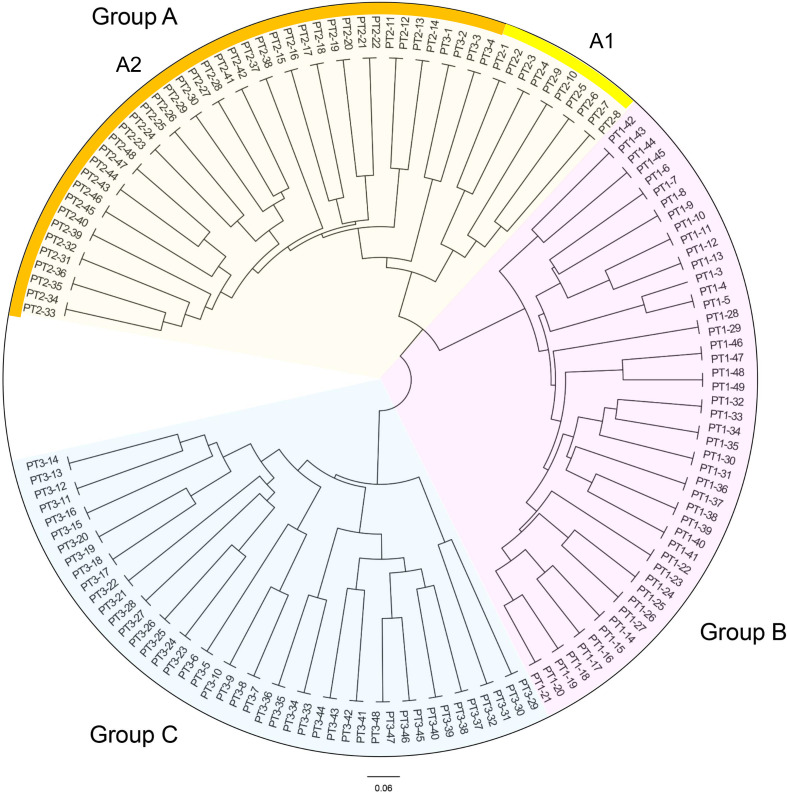
UPGMA dendrogram of the 3 C*. putoensis* generations population.

Based on the genetic distances, the UPGMA dendrogram of the three generations of *C. putoensis* populations is presented in **Figure 3**. As depicted, the 143 samples are classified into three groups designated A, B, and C. Group A encompasses 52 individuals, comprising all 48 individuals from the second generation and four from the third generation (1, 2, 3, and 4). This group can be further divided into two subgroups, A1 and A2, with subgroup A1 consisting of individuals numbered 1 through 10 from the second generation, and subgroup A2 representing the remaining individuals. Specifically, the F3 generation 1-4 was clustered in Group A. Group B consists of 47 individuals, representing the entire first generation. Within this group, individuals numbered 42, 43, 44, and 45 form a cluster, indicating a closer genetic relationship. Group C comprises 44 individuals from the third generation, and individuals PT3-29 to PT3-32 in group C form a cluster.

### Mating system

The mating system parameters were estimated for six family populations of the second generation of *C. putoensis* (**Table 5**). The results indicated that the overall multilocus outcrossing rate (*t_m_*) for the population, estimated from 15 loci, was 1.000, and the single-locus outcrossing rate (*t_s_*) was 0.871, with *t_m_*- *t_s_* being 0.129. These results suggest that biparental outcrossing is predominant in the population, albeit with a low proportion of mating among relatives. The number of effective pollen donors (*N_ep_*) within the second-generation *C. putoensis* family populations was relatively low, at only 4.00. The difference between single-locus (*r_p(s)_*) and multilocus (*r_p(m)_*) paternity correlations, which reflects the relationship between biparental relatedness and the mating population structure, was 0.064, greater than zero, indicating that only a tiny fraction of pollen donors was related, and there is a selective force within the population that favors inbreeding.

**Table 5:** Mating system parameters for 6 families of *C. putoensis*.

Family	Number of progeny	Multilocus outcrossing rate(*t*_m_)	Singlelocus outcrossing rate(*t*_s_)	Difference of outcrossing rates(*t*_m_-*t*_s_)	Multilocus correlation of paternity(*r*_p(m)_)	Singlelocus correlation of paternity(*r*_p(s)_)	Number of effective pollen donors(*N*_ep_)
Z3	30	1.000(0.000)	0.880(0.043)	0.120(0.043)	0.202(0.067)	0.171(0.036)	4.95
Z5	29	1.000(0.000)	0.920(0.037)	0.080(0.037)	0.249(0.100)	0.241(0.058)	4.02
Z6	30	1.000(0.000)	0.903(0.043)	0.097(0.043)	0.179(0.100)	0.187(0.051)	5.59
Z7	26	1.000(0.000)	0.918(0.035)	0.082(0.035)	0.063(0.053)	0.133(0.030)	15.87
Z9	25	1.000(0.000)	0.751(0.058)	0.249(0.058)	0.211(0.068)	0.140(0.027)	4.74
Z10	29	1.000(0.000)	0.892(0.053)	0.108(0.053)	0.299(0.077)	0.264(0.066)	3.34
Total	169	1.000(0.000)	0.871(0.027)	0.129(0.027)	0.250(0.048)	0.314(0.052)	4.00

The single-locus outcrossing rate (*t_s_*) varied among the six families, with *t_s_* ranging from 0.751 (family Z9) to 0.920 (family Z5). The range of the biparental inbreeding coefficient (*t_m_*-*t_s_*) was from 0.080 (families Z5) to 0.249 (family Z9), where the six families had positive biparental inbreeding coefficients, suggesting the occurrence of inbreeding. The paternity correlation (*r_p_*) among the families showed considerable variation, ranging from 0.063 to 0.299, with the number of effective pollen donors ranging from 3.34 to 15.87, indicating inconsistent levels of paternity correlation across families, with family Z7 having the highest and families Z10 the lowest.

## Discussion

Rare and endangered plant species are facing high danger of extinction, necessitating immediate and effective conservation strategies to avert their disappearance. The three major techniques for plant conservation are *in situ* conservation, *ex situ* conservation, and reintroduction (Ren et al., 2014). *In situ* conservation is defined as “the maintenance and recovery of viable populations of species in their natural surroundings” (Xu and Zang, 2023). *In situ* conservation generally encompasses the creation of nature reserves, national parks, wildlife sanctuaries, and other conservation areas. Such conservation is crucial for preserving the natural ecological processes of species and maintaining the pristine ecological support systems, including climate and soil conditions. Nonetheless, *in situ* conservation alone is inadequate for population recovery for species at risk due to environmental factors. *Ex situ* conservation involves maintenance and breeding of plants under partially or wholly controlled conditions in specific areas outside their natural habitat. This approach alleviates the pressures on individual organisms due to competition for resources and space by offering the necessary conditions for secure living and reproduction. *Ex situ* conservation is essential when the habitats of rare and endangered plants are severely degraded or fragmented (Huang and Zhang, 2012). Reintroduction is defined as “the deliberate establishment of individuals from a species into an area and/or habitat where it has been extirpated, with the specific aim of establishing a viable, self-sustaining population for conservation purposes” (Xu and Zang, 2023). Reproduction is pivotal in the renewal and maintenance of plant species populations. Many rare and endangered plant species face reproductive challenges, such as the unsynchronized development of stamens and pistils, pollen abortion, and embryo sac abortion. These disturbances can lead to a decline in species fitness and abundance, and in severe cases, even extinction. Artificial propagation of rare and endangered plant species can be used for *ex situ* conservation and reintroduction. The seeds of *C. putoensis* have a thick and hard seed coat, which results in poor germination, with a nursery emergence rate of only 2.5% (Meng et al., 2004). The overlapping period for the female and male flowers of *C. putoensis* is a mere 6 days, making the pollination period very brief. The pollen germination rate is also low, and self-pollination typically leads to seed abortion. Furthermore, frequent strong winds on Putuo Island cause the fruits to be blown off before they mature. Due to the aforementioned factors, no naturally regenerated seedlings have been observed around the mother tree to this day. Nevertheless, through seed collection from the mother tree and artificial propagation, we have successfully established a substantial population of the first, second, and third filial generations of *C. putoensis.*

Genetic diversity encompasses the total genetic variation within a species population, playing a crucial role in the species’ endurance over time and mirroring its capacity to adapt to environmental changes and evolve (Vinson et al., 2018). In this study, we used microsatellite markers to investigate the genetic diversity within the three-generation populations of *C. putoensis*. The results indicate that the genetic diversity level of the three generations of *C. putoensis* populations is moderate, with an average expected heterozygosity of 0.617, and there is a slight increase in expected heterozygosity with the increase of generations. *C. putoensis* is a species of the genus *Carpinus* in the family Betulaceae. Plants in the Betulaceae family play a significant role in the flora of the northern temperate zone, and species within this genus are widely distributed, exhibiting pollen characteristics that are adapted for wind pollination. Reports have indicated considerable variation in genetic diversity among species in the Betulaceae family. Within the genus *Betula* of the Betulaceae family, both *Betula pendula* L. and *Betula pubescens* Ehrh. exhibit relatively high levels of genetic diversity (Vetchinnikova and Titov, 2023). However, there is considerable variation in genetic diversity among different populations of *Betula pendula* var. *carelica*. In the Karelian region of northwestern Europe, the expected heterozygosity of *Betula pendula* var. *carelica* populations range from 0.38 to 0.68. Furthermore, in populations of *Betula pendula* var. *carelica*, there is a situation where the expected heterozygosity is higher than the observed heterozygosity, suggesting that there may be a relatively high number of homozygotes in the population. This could reduce gene flow and potentially increase the risk of population decline (Vetchinnikova and Titov, 2023). Additionally, within the genus *Corylus* of the family Betulaceae, K. Gürcan conducted a genetic diversity analysis of *Corylus avellana* L. from 14 different regions and found that the average expected heterozygosity among 50 genotypes of European filbert was 0.76, indicating a relatively high level of genetic diversity (Gürcan et al., 2010).

However, compared to other rare and endangered plants in various regions, the expected heterozygosity of *C. putoensis* remains relatively high. For instance, the expected heterozygosity of *Paeonia decomposita* (Wang, 2020) is 0.405, the Nei’s gene diversity of *Rhododendron protistum* (Wu et al., 2014) is 0.24, and that of *Cycas hongheensis* (He et al., 2023) ranges from 0.128 to 0.360. Additionally, four endangered plants on Mediterranean islands in Greece, *Aethionema retsina*, *Allium iatrouinum*, *Convolvulus argyrothamnos*, and *Saponaria jagelii* (Kougioumoutzis et al., 2021), have expected heterozygosities of 0.290, 0.319, 0.322, and 0.254, respectively. Research has demonstrated that the genetic diversity and structure of species are influenced by various factors, including distribution range, reproductive modes, evolutionary history, climate, and human interference, so endangered plants do not necessarily show low genetic diversity (He et al., 2024). *Pityopsis ruthii* is a federally endangered herbaceous perennial endemic to the Hiwassee and Ocoee Rivers in southeastern Tennessee, and it exhibits a relatively high level of expected heterozygosity (0.65), indicating considerable genetic diversity (Hatmaker et al., 2018). A population genomic study of *Mimosa catharinensis*, a narrow endemic and critically endangered plant species, identified 1,497 unlinked SNP markers and revealed a moderate genetic diversity (unbiased expected genetic diversity(*uH*_E_)=0.205) (Teixeira and Huber, 2021). Additionally, the endangered medicinal plant *Ferula sinkiangensis* (Apiaceae) exhibits intermediate genetic diversity (nucleotide diversity, π = 0.086), as assessed using 61,344 SNPs derived from 229 individuals (Wariss et al., 2025).

It is noteworthy that, based on AFLP markers and ITS sequences, the *C. tientaiensis*, also a member of the *Carpinus* genus, has been found to possess medium to high levels of genetic diversity (Zhao et al., 2023, 2024). Despite having a very small number of natural individuals, it is listed as critically endangered on the IUCN Red List of Threatened Species. This indicates that wind-pollinated reproductive characteristics facilitate the increase of genetic diversity within populations; however, climate change also significantly impacts the success rate of wind pollination. The previously effective reproductive systems are unable to adapt to the drastic environmental changes currently occurring, which may be the primary reason why both *C. putoensis* and *C. tientaiensis*, despite their high genetic diversity, are still facing the threat of extinction.

Traditional island biogeography theory suggests that island populations isolated from mainland sources typically exhibit reduced genetic variation compared to their mainland counterparts (Lindgren and Cousins, 2017; Patiño et al., 2017). This expectation has been empirically supported across various taxonomic groups (Frankham, 1997; Harradine et al., 2015; Jennings et al., 2016; Robinson et al., 2016). Island populations may experience the founder effect, which occurs when a population is established by a small number of individuals, leading to an initial reduction in genetic diversity (Kivisild, 2013). Prolonged geographical isolation can lead to restricted gene flow, further diminishing genetic diversity. However, several recent studies have found that, due to the role of islands as climate refuges during glacial periods (Fernandez-Mazuecos and Vargas, 2011; García-Verdugo et al., 2015) and the repeated colonization of islands by marine plants (Takayama et al., 2005), marine plants may exhibit higher genetic diversity than their continental relatives. There is a growing belief that the lower genetic diversity in island populations is not always a foregone conclusion (Patiño et al., 2017), and that genetic diversity is more likely to be strongly influenced by population size and historical factors, such as the time since population establishment and past bottlenecks (Hamabata et al., 2019).

The mating system has long been considered a significant factor affecting the genetic structure of plant populations (Whitehead et al., 2018). It governs the transmission and perpetuation of genes from the gametophyte between two generations, playing an essential role in the genetic structure of plant populations (Devaux et al., 2014; Barrett and Harder, 2017; Li et al., 2022). In *C. putoensis*, both multilocus and single-locus outcrossing rates are notably elevated, indicating a high level of outcrossing. The anthesis of male and female flowers in *C. putoensis* is not entirely synchronized, and their overlapping period is short, which affects pollination and fertilization. This may be the main reason for the high outcrossing rate in *C. putoensis* populations (Meng et al., 2004). The effective number of pollen donors for the entire population is 4.00, which is at a low level, with only the Z7 family having an effective number of pollen donors of 15.87. The scarcity of effective pollen donors aligns with our phenological observations, where the asynchrony between the male and female flowering phases often leads to the failure of individual plants’ pollen to achieve fertilization. Furthermore, in our seed collection efforts, it has been observed that most plants are incapable of producing viable seeds, attributed to the fact that during the reproductive phase, female flowers fail to receive pollen from male flowers. In fact, periodic seed production is commonly observed in tree species, often manifesting as alternating years of fruiting. However, with the *C. putoensis*, if the number of seeds produced is high in a given year, the species will not produce seeds for several consecutive years thereafter. Typically, it takes us several years to harvest and accumulate enough seeds for artificial propagation. The mechanism by which such a limited number of pollen donors can contribute to a slight increase in the genetic diversity across generations of *C. putoensis* remains unclear. It is possible that, despite the limited number of pollen donors, heterozygous individuals with higher survival or reproductive success may play a significant role in this process.

It is worth noting that the molecular markers utilized in this study were EST-SSRs derived from transcriptome data. Following the Hardy-Weinberg equilibrium test, 10 of 15 primer pairs showed significant deviation from genetic equilibrium (**Supplementary Table S3**), indicating that these 10 primer pairs are non-neutral molecular markers. Given the genetic diversity characteristics of island endangered plants (Hamabata et al., 2019), our estimates of genetic diversity for the first, second, and third filial generations of *C. putoensis* may be higher than the actual situation. However, more precise genetic diversity analyses rely on the application of neutral molecular markers at the whole genome level. Non-neutral SSRs can significantly impact genetic diversity analyses as they do not adhere to the neutrality assumptions that underpin many metrics and interpretations in population genetics. Neutral molecular markers provide valuable insights into parameters such as genetic diversity within populations, genetic differentiation among populations, inbreeding, and demographic events, albeit with limited insight into adaptive evolution and evolutionary potential (Kirk and Freeland, 2011). With advancements in NGS sequencing technology, we are increasingly capable of developing non-neutral markers by targeting genetic regions directly influenced by natural selection, enabling an increasing number of studies to directly investigate natural selection and local adaptation in natural populations (Kirk and Freeland, 2011). Non-neutral EST-SSRs may identify gene regions associated with adaptability, as high-frequency alleles may result from natural selection. Recently, there has been growing interest in the comparison and application of neutral and non-neutral (adaptive) genetic variations. Neutral molecular markers are deemed effective and significant indicators, yet adaptive molecular markers complement neutral ones significantly (Chung et al., 2023). The use of neutral molecular markers alone has limitations in forecasting the adaptive potential of populations (Teixeira and Huber, 2021), and an exclusive reliance on adaptive molecular markers to craft conservation strategies might lead to an underestimation of the number of populations needing conservation. In this study, the genetic diversity of the offspring of *C. putoensis* may be overestimated, thus warranting the implementation of more extensive conservation measures for populations. Future efforts will prioritize offspring with higher genetic diversity, expand *ex situ* conservation trials, and identify more suitable habitats for *C. putoensis* to ensure its long-term conservation and utilization.

Climate change and human disturbance are significant factors leading to the endangerment of plant species. If no action is taken to address the critical state of this species, *C. putoensis* may meet the same fate as other species that have become extinct on Earth (Mace et al., 2008; Pimm et al., 2014; Cowie et al., 2022). A report published by Kew Gardens in the UK states that two-fifths of the world’s plant species are at risk of extinction (Antonelli et al., 2023). *C. putoensis* was once on the verge of extinction with only one mature tree remaining and has been the focus of genetic conservation efforts by the local forestry administration since 1981. Through *in situ* conservation, *ex situ* conservation, artificial propagation, many *C. putoensis* saplings have been successfully cultivated, temporarily averting the risk of extinction. In 2014, 35 individuals were reintroduced on Buddha’s Summit Mountain, with 300 trial-planted in 2016 and 1000 in 2018. Over 30 *ex situ* conservation trial sites have been set up across China’s 7 provinces and cities. Our study revealed that the *C. putoensis* population has adequate genetic diversity, however, reproductive traits and the unique environmental conditions of the island impede its natural regeneration. This highlights the pivotal role of artificial promotion in increasing the population size of *C. putoensis*, which is crucial for the future conservation and genetic diversity enhancement of the species.

## Data Availability

The data presented in the study are deposited in the NCBI repository, accession number PRJNA1224840.
