# Supplementary material for: Revealing that artificial reproduction promotes increased genetic diversity between generations in Carpinus putoensis
Source: Front Plant Sci. 2025 Feb 27;16:1494694. doi: 10.3389/fpls.2025.1494694 (PMC11903413; doi:10.3389/fpls.2025.1494694)
Supplement: Supplementary file 1 [file Table1.docx]

**Supplementary Table S1 ANOVA of *H_o_* among three generations**

| **Source of Variation** | **SS** | **df** | **MS** | **F** | **P-Value** |
| --- | --- | --- | --- | --- | --- |
| Between Groups | 0.015 | 2.000 | 0.008 | 0.056 | 0.945 |
| Within Groups | 5.750 | 42.000 | 0.138 |  |  |
| Corrected Total | 5.765 | 44.000 |  |  |  |

**Supplementary Table S2 Post Hoc Tests for ANOVA of *H_o_* among three generations**

| Generation(I) | Generation(J) | Mean Difference (I-J) | Standard Error | Significance | 95% Confidence Interval | |
| --- | --- | --- | --- | --- | --- | --- |
|  |  |  |  |  | Lower Bound | Upper Bound |
| F1 | F2 | 0.002 | 0.143 | 1.000 | -0.361 | 0.365 |
|  | F3 | -0.038 | 0.136 | 0.989 | -0.385 | 0.309 |
| F2 | F1 | -0.002 | 0.143 | 1.000 | -0.365 | 0.361 |
|  | F3 | -0.040 | 0.127 | 0.985 | -0.363 | 0.282 |
| F3 | F1 | 0.038 | 0.136 | 0.989 | -0.309 | 0.385 |
|  | F2 | 0.040 | 0.127 | 0.985 | -0.282 | 0.363 |

**Supplementary Table S3 The Hardy-Weinberg equilibrium test for 15 pairs of SSR primer sites in *C. putoensis***

| **Locus** | **L3** | **L7** | **P1** | **P3** | **P5** | **P7** | **P8** | **P9** | **P11** | **P12** | **P13** | **P14** | **P15** | **P16** | **P17** |
| --- | --- | --- | --- | --- | --- | --- | --- | --- | --- | --- | --- | --- | --- | --- | --- |
| HW | *** | *** | NS | *** | *** | *** | *** | NS | *** | NS | ND | *** | ND | *** | *** |

Note: *** indicates an extremely significant result at the 0.1% significance level; NS means Not Significant; ND means Not Determined.
